# Supplementary material for: Role of SALL4 in HER2+ Breast Cancer Progression: Regulating PI3K/AKT Pathway
Source: Int J Mol Sci. 2022 Oct 31;23(21):13292. doi: 10.3390/ijms232113292 (PMC9655635; doi:10.3390/ijms232113292)
Supplement: Supplementary file 1 [file ijms-23-13292-s001.zip › ijms-1964532-supplementary.pdf]

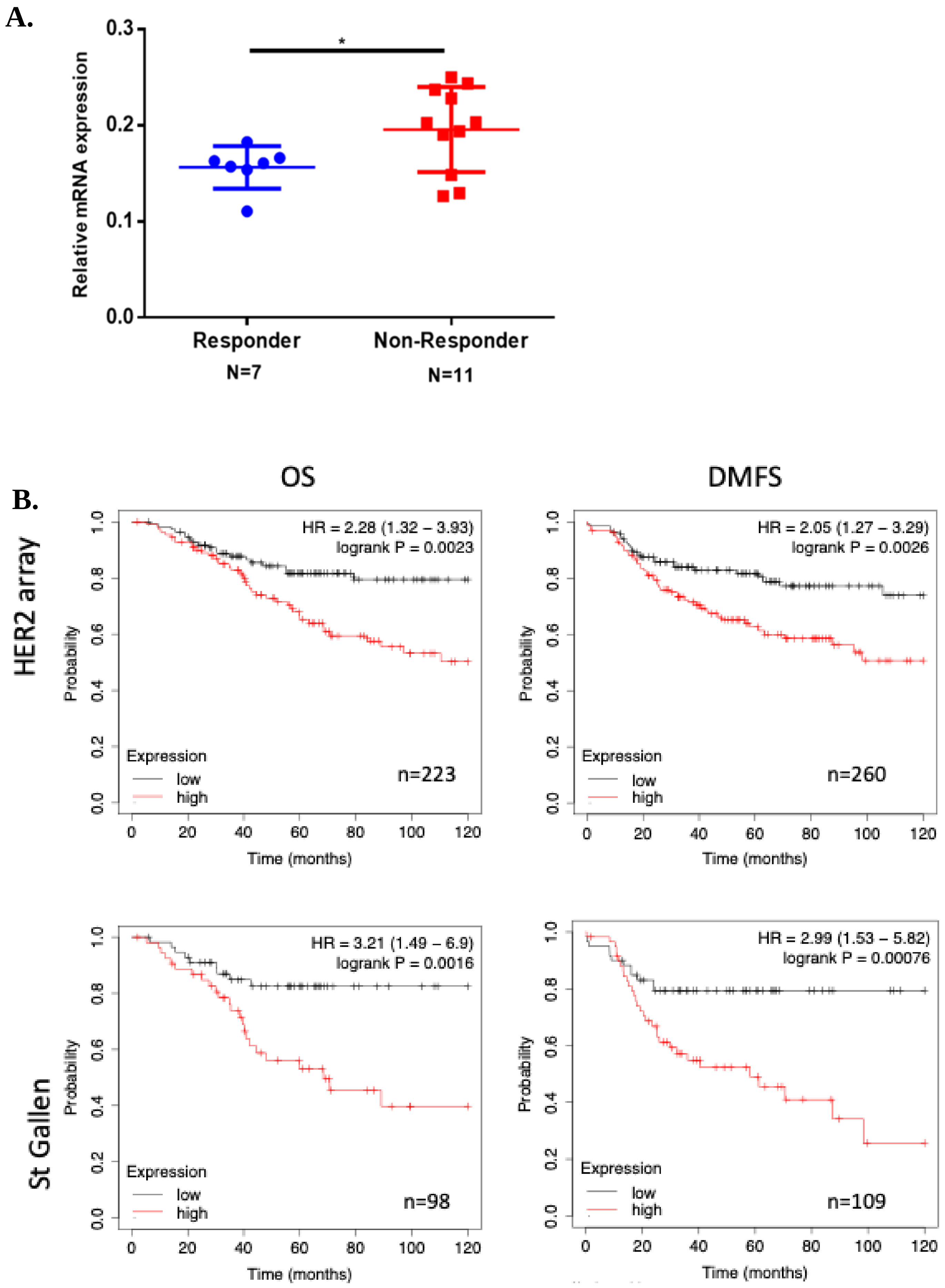

Supplementary Figure S1. **Expression of SALL4 in HER2+ breast cancer samples.** (A) Relative expression of *SALL4* in HER2+ breast cancer samples from responders and non-responders to trastuzumab treatment. Determination performed by RT-qPCR using GAPDH as endogenous gene. Student's t-test compared the results. *p-value $\leq$ 0.05. SALL4 prognostic value in breast cancer patients. (B) In-silico overall survival (OS) left, and distant metastasis-free survival (DMFS) right of SALL4 in HER2+ subtype. The top figures correspond to samples from the HER2 array database and the bottom figures correspond to samples from the St Gallen database, both included in the in the Kaplan-Meier Plot tool. Patients were split by median value and the follow-up was 120 months. the Hazard ratio (HR) and p-value are indicated for each analysis at the top right of teach box.

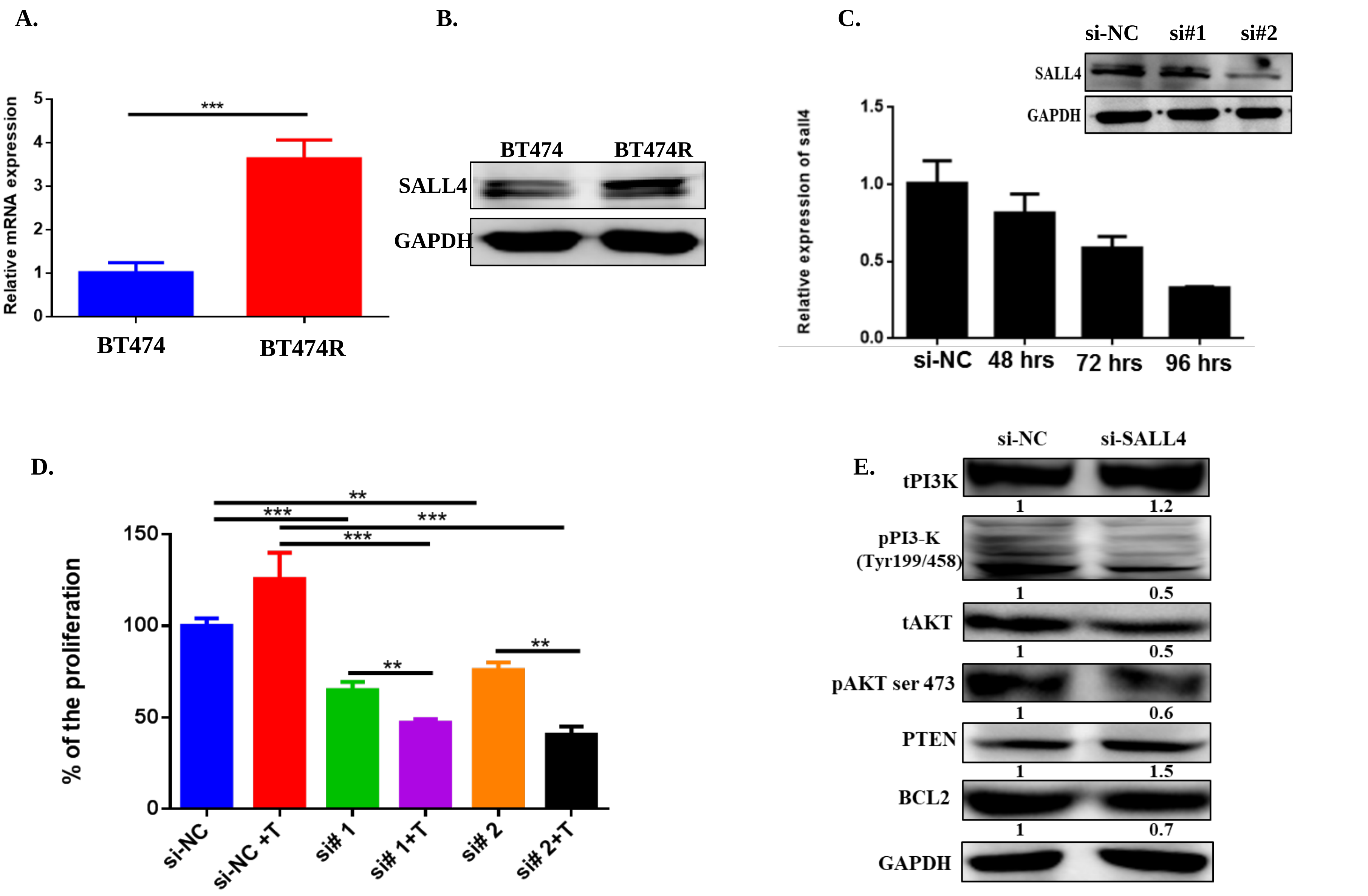

Supplementary Figure S2. Effect of SALL4 in BT474R cell line. (A) Relative expression of SALL4 was determined in BT474 HER2+ BC cell line parental and trastuzumab acquired resistance BT474R HER2+ BC cell line. (B) SALL4 protein expression was determined by western blot in mentioned cell lines. Student's t-test was used to analyse the significant differences. ***P ≤ 0.001. (C) Relative expression of SALL4 using specific silencers (si#1 and si#2) at mRNA and protein level in BT474 cell line with acquired resistance to trastuzumab (BT474R). (D) BT474R proliferation determined by WST assay with and without the presence of trastuzumab (T). The graph shows the results of three independent experiments. The experiments were carried out to 7 days. Student's t-test was used to compare the results. ** p-value ≤ 0.01 and *** p-value ≤ 0.001. (E) PI3K/AKT pathway associated proteins are analyzed by Western blot after down-regulation of SALL4 in BT474R. Expressions of PI3K/AKT pathway proteins, including PI3K, phospho-PI3 kinase (Tyr458 and Tyr199), phospho-Akt (Ser473), PTEN, BCL2 were shown. GAPDH expression was used as an internal control. si-NC: silencer negative control, si#1: silencer 1, si#2: silencer 2, T: trastuzumab.

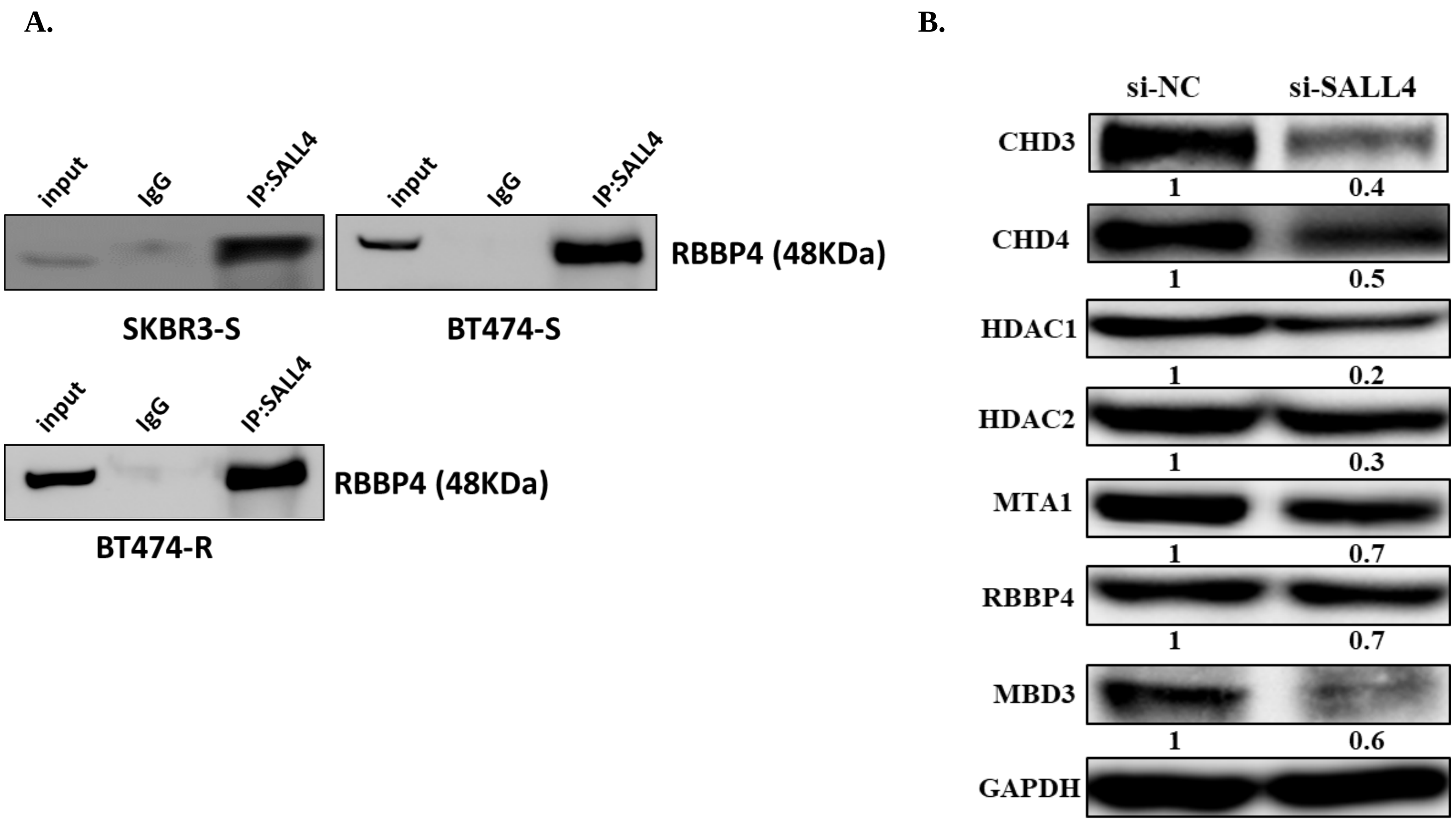

Supplementary Figure S3. **NuRD complex in HER2+ acquired resistance cell line BT474R**. (A) Co-Immunoprecipitation western blot revealing the physical interaction between SALL4 and RBBp4 in SKBR3 and BT474 parental HER2+BC cell lines and BT474R trastuzumab acquired resistance HER2+BC cell line. (B) Expression of the NuRD complex in HER2+ acquired resistance cell line BT474R after silencing SALL4. si-NC: silencer negative control, si-SALL4: silencer of SALL4.

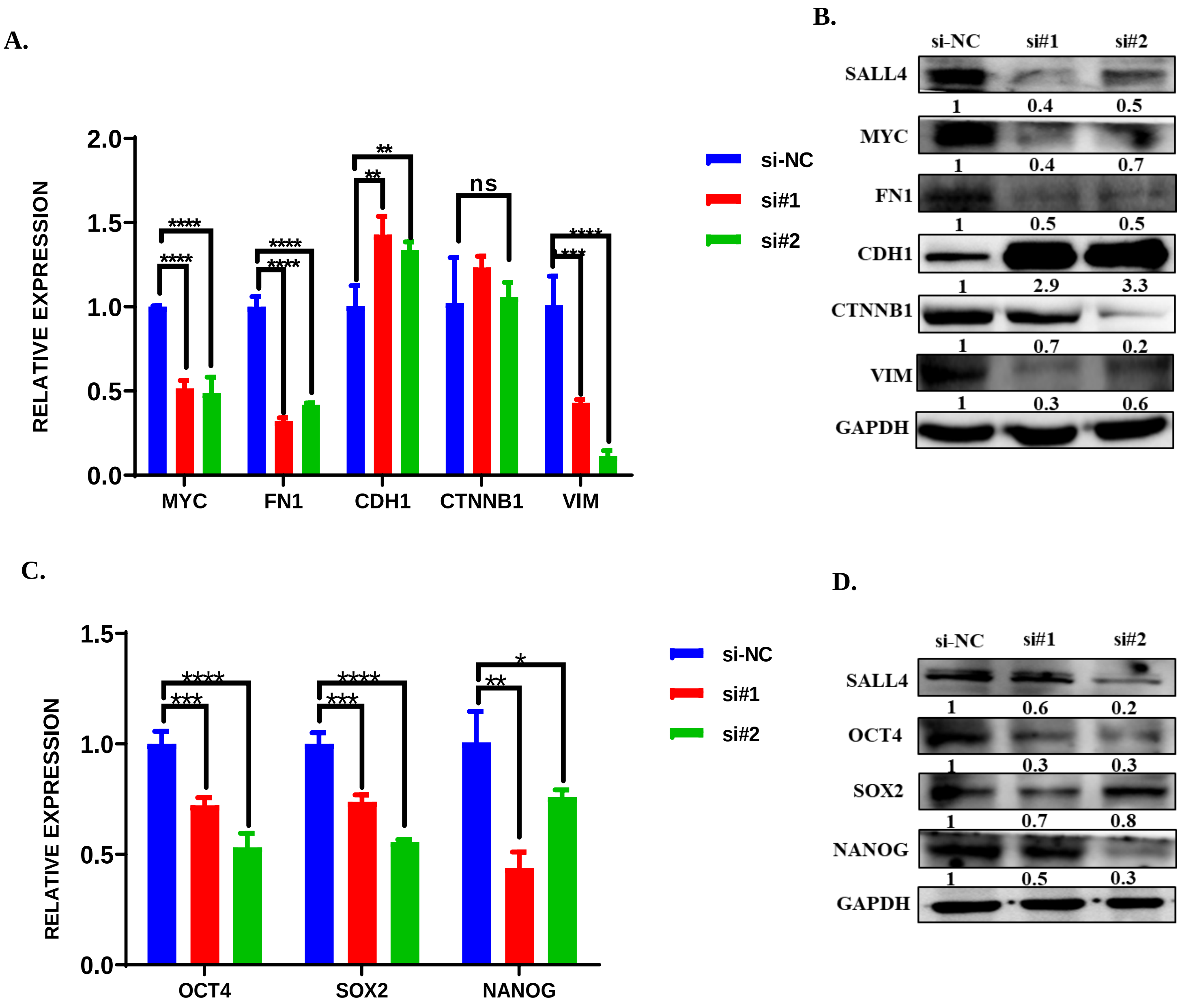

Supplementary Figure S4. **Modulation of EMT and stemness processes by SALL4 silencing in BT474R cells.** Reducing SALL4 expression in HER2+ trastuzumab resistance cell line (BT474R) effect in both EMT process and maintain stemness of the cells in both mRNA and protein levels. si-NC: silencer negative control, si#1: silencer 1, si#2: silencer 2. * p-value $\leq$ 0.05 ** p-value $\leq$ 0.01 *** p-value $\leq$ 0.001 and **** p-value $\leq$ 0.0001

Supplementary Table S1: List of antibodies

Antibodies	References
SALL4	24500-1-AP, Proteintech
Total PI3K	4292, Cell Signaling
Phospho PI3K Tyr-458 and Tyr-199	4228, Cell Signaling
Total AKT	9272, Cell Signaling
Phospho AKT Ser-473	9271, Cell Signaling
PTEN	9188, Cell Signaling
Bcl-2	15071, Cell Signaling
GAPDH	MA5-15738, Thermo Scientific™
NuRD Complex Antibody Kit	8349, Cell Signalling
MYC	13987, Cell Signalling
FN1	ab32419, Abcam
E-cadherin	610181, BD Biosciences
Beta-catenin	610153, BD Biosciences
Vimentin	550513, BD Biosciences
SOX2	3579, Cell Signaling
NANOG	4903, Cell Signaling
OCT4	2840, Cell Signaling
Anti-rabbit IgG, HRP-linked (Secondary antibody)	7074, Cell Signalling
